# Association of Fibrinogen With Ischemic Stroke: A Systematic Review and Meta-Analysis

**DOI:** 10.7759/cureus.34335

**Published:** 2023-01-29

**Authors:** Manoj K Prasad, Sujeet Marandi, Brajesh Mishra, Rishi T Guria, Amit Kumar, Hirendra Birua, Hemant N Ray, Ajit Dungdung, Divakar Kumar, Shubham Maitra

**Affiliations:** 1 Internal Medicine, Rajendra Institute of Medical Sciences, Ranchi, IND; 2 Pulmonary Medicine, Rajendra Institute of Medical Sciences, Ranchi, IND; 3 Neurology, Rajendra Institute of Medical Sciences, Ranchi, IND; 4 Pediatric Surgery, Rajendra Institute of Medical Sciences, Ranchi, IND; 5 Cardiology, Rajendra Institute of Medical Sciences, Ranchi, IND

**Keywords:** ischemic stroke, meta-analysis, systematic review, odds ratio, fibrinogen

## Abstract

Validation of a risk factor in a multifactorial disease like ischemic stroke is necessary to practice precision medicine. Many risk factors have been attributed to causing ischemic stroke but contribute very little to it. There are many risk factors that need to be validated, and fibrinogen is one such risk factor. Using a meta-analysis technique, we investigated fibrinogen as a risk factor for ischemic stroke.

We searched the computerized databases such as PubMed, Google Scholar, and Cochrane to explore articles on ischemic stroke. Pooled odds ratios (ORs) with 95% confidence intervals (CIs) were calculated using a random effects model.

A total of 10 case-control studies with 6877 cases and 7219 controls were included in the study that match inclusion and exclusion criteria. The Asiatic population was portrayed in four studies, whereas the Caucasian population was portrayed in six studies. Under the recessive model, an elevated level of serum fibrinogen is linked to an increased risk of ischemic stroke as shown by pooled odds ratio (OR: 1.47, 95% CI: 1.19-1.76, I^2^ = 78.3%, P = 0.000).

Our meta-analysis concluded that a high level of fibrinogen is associated with an increased risk of ischemic stroke.

## Introduction and background

Ischemic stroke is a condition due to sudden deprivation of blood supply to a part of the brain leading to a focal neurological deficit. It is caused by thrombotic or embolic occlusion of cerebral arteries and is more common than hemorrhagic stroke. Looking at the worldwide scenario, it is the second major cause of demise and disability, of which most of the cases are from low- and average-income countries [1]. About 60%-80% of ischemic strokes are associated with hypertension, diabetes mellitus, hyperlipidemia, smoking, carotid stenosis, atrial fibrillation, and valvular heart disease [2]. There were 13.7 million new stroke cases globally in 2016, and ischemic stroke cases constituted 87% of them [1]. Atherosclerosis is an important pathophysiological process that contributes to stroke and cardiovascular events. The above-mentioned risk factors are important in the causation of stroke, but as stroke is multifactorial, the contribution of each of these factors is still a matter of debate. According to the literature, hypertension is the second most important risk factor for stroke after age, and a person with hypertension is three to four times more likely to have a stroke [3].

There are many inflammatory markers whose levels tend to increase after an acute attack of ischemic stroke. Fibrinogen is one such important inflammatory marker. It is an acute-phase protein that is seen to rise after stroke and myocardial infarction [4]. An early sign of atherosclerosis is associated with increased fibrinogen levels in asymptomatic patients [5].

The rationale behind taking up this study is that there has been no isolated systematic review and meta-analysis in the literature so far considering the association of fibrinogen with an increased risk of ischemic stroke. One meta-analysis has been done considering fibrinogen as a risk factor in cardiovascular disease, in which pooled odds ratio (OR) of 2.3 (CI: 1.9 to 2.8) was found [4]. One of the main factors associated with both ischemic stroke and ischemic cardiovascular disease is atherosclerosis. As early atherosclerosis is associated with increased fibrinogen levels shown in recent studies, fibrinogen levels may be associated with ischemic stroke. As fibrinogen level test can be measured easily and is not expensive, the increased fibrinogen level can be used as an adjuvant test for ischemic stroke in low- and average-income countries where the resource is poor, thereby helping the health care providers to take early and optimal steps in the prevention and treatment of ischemic stroke. Fibrinogen-associated ischemic complications of atherosclerosis can be identified readily; therefore, non-pharmacological and pharmacological measures to reduce serum fibrinogen levels can be instituted at the earliest for secondary prevention [6]. The purpose of this meta-analysis is to screen, analyze, and amalgamate all the available studies for pooled data to answer the question of whether increased fibrinogen level is associated with an increased risk of ischemic stroke.

## Review

Methods

Search Strategy

The relevant studies were searched individually by MKP and SM on PubMed, Cochrane database, and Google Scholar from 1991 to April 2022. The search strategy consisted of the following search term: Blood OR serum OR plasma AND Fibrinogen AND ischemic stroke. The search was performed by two independent reviewers. During the search, any discrepancies were resolved by mutual discussion among us and with other authors involved in this meta-analysis. Studies reporting the association of fibrinogen with ischemic stroke were identified, and their references were also screened.

Inclusion and Exclusion Criteria

Studies reporting the association of fibrinogen as a risk factor for ischemic stroke, studies reporting sufficient data to pooled OR, adult patients aged more than 18 years, and full-text articles published in English were included. Articles not in English language, editorials, letters to editors, conference proceedings, and abstracts were excluded.

Study Selection

Each article was scrutinized individually by two authors to determine whether they meet the incorporating criteria. The differences in opinion were resolved by mutual discussion among us and with other authors.

Data Extraction

Relevant quantitative data were drawn out by two writers (MKP and SM) in the form of first author name, year of publication, the cut-off for fibrinogen value, OR with lower and upper confidence intervals (CIs), the number of cases and the number of controls, mean age, sex, ethnicity, type of blood sample for fibrinogen (serum/plasma), methods used to test for fibrinogen, and other variables mentioned in the demographic profile of each study (hypertension, diabetes, alcohol, smoking, total cholesterol, serum triglyceride, etc.).

Assessment of the Quality of Study

As all the selected studies are of case-control study type, the standard of the different studies was assessed individually by MKP and SM using the Newcastle-Ottawa scale. Each study was assessed for the adequacy of case definition, the representativeness of cases, the selection of control, the definition of control, the ascertainment of exposure, the same method of ascertainment for cases and control, the non-response rate, and the assessment of outcome. Each end result was further classified as good, fair, or poor as per Agency for Health Care Research and Quality (AHRQ) standards.

Statistical Analysis

The software STATA version 13 (StataCorp LP, College Station, TX) was used for the analysis of data. Z-test was done to find out the pooled effect size using the random effect model, and the I^2^ test was used to look for the heterogeneity among the studies. A forest plot was computed to find out the pooled OR. Meta-regression analysis was done for each variable (age, sex, alcohol, hypertension, diabetes mellitus, smoking, serum cholesterol, triglyceride, cut-off for blood fibrinogen level, sample size, ethnicity, body mass index or BMI, high-density lipoprotein or HDL, and low-density lipoprotein or LDL) to look for the cause of heterogeneity. Publication bias of the studies was assessed by drawing a funnel plot of studies included in this meta-analysis. The unavailable data were marked as NA (not available).

Result

Study Selection

**Figure 1:**
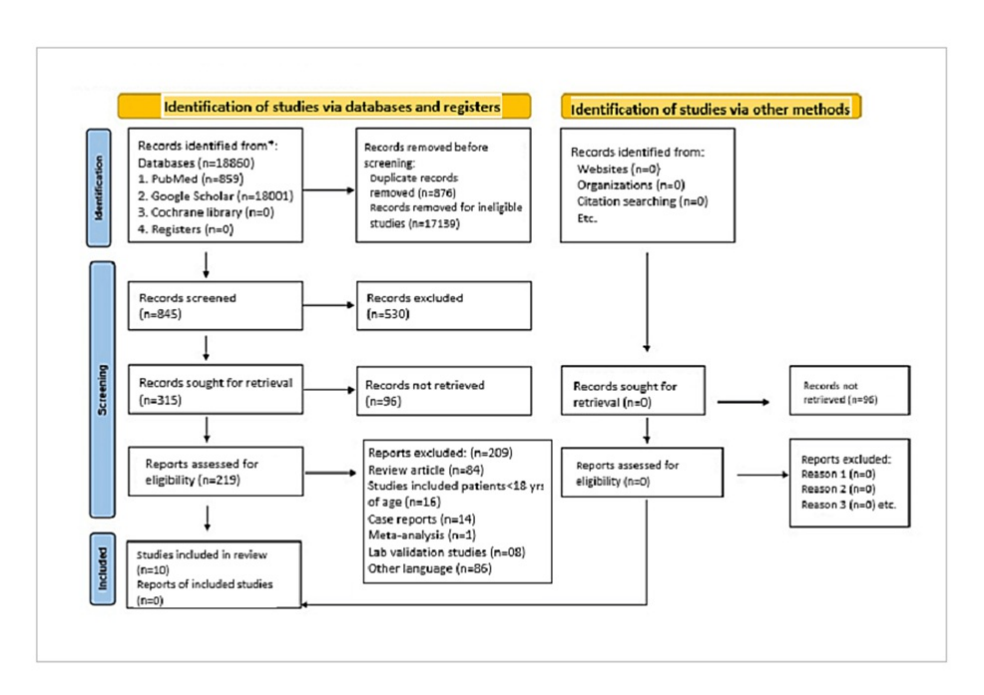
PRISMA flow diagram of the study PRISMA: Preferred Reporting Items for Systematic Reviews and Meta-Analyses.

Upon preliminary search, a total of 18,860 studies were retrieved through a computerized database. Irrelevant articles were excluded by going through the titles and abstracts, and 315 studies were sought for retrieval out of which 96 studies could not be retrieved. A total of 219 studies were assessed for eligibility, and 209 studies were excluded; finally, 10 studies that fulfilled the inclusion criteria were included in our meta-analysis [7-16]. All these studies were of case-control design. A flow diagram is drawn in Figure 1 depicting the procedures of literature search and inclusion of studies as per the latest Preferred Reporting Items for Systematic Reviews and Meta-Analyses (PRISMA) 2020 protocol.

Characteristics of the Study

A total number of 6877 cases and 7239 controls were incorporated into this study. The general characteristics of the study subjects are tabulated in Tables 1, 2.

**Table 1:** General characteristics of the patient Note: In the studies where two values for fibrinogen level have been given [8,10,11,13,15], the higher value is for the case and the lower value is for the control as the cut-off value for the fibrinogen level was not provided in the study. gm/l: Gram per liter; OR: Odds ratio; CI: Confidence interval; C: Caucasian; A: Asian; CCS: Case-control study. Source: References [7-16].

S. No.	Author	Year	Study Design	Country	Ethnicity	Fibrinogen Cut-off (gm/L)	Sample	Fibrinogen Assay Method	OR	Lower CI	Upper CI	Case	Control	Age All	Sex (Male in %)
1	Qizilbash et al. [7]	1991	CCS	England	C	3.6	Serum	Ellis and Stransky (modified)	1.78	0.91	3.48	104	241	67.65	66.4
2	Kristensen et al. [8]	1998	CCS	Sweden	C	3.12, 2.34	Plasma	Thrombin reaction time kit from biomatrix	11.25	3.27	38.69	102	41	35.9	58.27
3	Bots et al. [9]	2002	CCS	Europe	C	3.4	NA	NA	1.37	1.14	1.65	138	521	62.7	76.4
4	Milionis et al. [10]	2005	CCS	Greece	C	4.3,3	Plasma	Clauss method	1.1	1.05	1.13	163	166	77.5	53.2
5	Zhang et al. [11]	2007	CCS	China	A	4.5,3.8	Plasma	Kit method	2.09	1.02	4.27	50	54	62.53	58.65
6	Jood et al. [12]	2008	CCS	Sweden	C	3.7	Plasma	Automated clot-rate assay	3.2	2.51	4.09	600	600	56	64
7	Prugger et al. [13]	2013	CCS	Europe (France, Ireland)	C	3.25,3.1	Plasma	ELISA (Diagnostica Stago, Asnières-sur-Seine, France)	1.53	1.03	2.28	95	190	55.55	100
8	Imran et al. [14]	2015	CCS	Indonesia	A	3.75	Plasma	von Clauss principle with Fibri-Prest Automate (Diagnostics Stago, Inc.).	2.28	1.28	4.07	107	94	39.5	42.8
9	Tao et al. [15]	2020	CCS	China	A	3.4,2.98	plasma	STAR evolution automatic blood coagulation analyzer (France, STAGO)	3.46	1.05	11.42	100	47	59	64.62
10	Karim et al. [16]	2020	CCS	China	A	3.01	Plasma	BN Prospec nephelometer analyzer (Siemens, UK)	1	0.84	1.19	5418	5285	55.6	47.36

**Table 2:** Different variables included in the study DM: Diabetes mellitus; HTN; Hypertension; BMI: Body mass index; kg/mt sq: Kilogram per meter square; Chol: Cholesterol; mmol/l: Millimole per liter; LDL: Low-density lipoprotein: HDL: High-density lipoprotein: NA: Not available. Source: References [7-16].

S. No	Author	Year	DM (%)	HTN (%)	Smoking (%)	Alcohol (%)	BMI (kg/m^2^)	Total Chol (mmol/l)	Triglyceride (mmol/l)	LDL (mmol/l)	HDL (mmol/l)
1	Qizilbash et al. [7]	1991	5.7	40	70.11	89.1	NA	NA	NA	NA	NA
2	Kristensen et al. [8]	1998	NA	NA	25.21	NA	24.95	5.35	1.45	NA	NA
3	Bots et al. [9]	2002	6.4	NA	33	NA	NA	6.2	NA	NA	NA
4	Milionis et al. [10]	2005	24.32	41.95	26.75	NA	25.8	5.36	1.69	3.41	1.18
5	Zhang et al. [11]	2007	12.5	33.65	21.15	12.5	NA	4.45	1.53	2.55	1.18
6	Jood et al. [12]	2008	12.25	48.17	28.5	NA	26.5	NA	NA	NA	NA
7	Prugger et al. [13]	2013	5.3	17.57	NA	NA	26.75	5.76	NA	NA	1.26
8	Imran et al. [14]	2015	9.45	44.3	38.32	NA	NA	5	1.44	2.81	NA
9	Tao et al. [15]	2020	27.21	54.42	38.1	25.85	NA	4.14	1.2	2.63	1.23
10	Karim et al. [16]	2020	9.24	17.13	NA	33.64	23.75	NA	1.7	2.42	NA

Six studies [7-10,12,13] having a study population of 2961 (case: 1202 and control: 1759) were done in the Caucasian population, and four studies [11, 14-16] having a study population of 11155 (case: 5675 and control: 5480) were done in the Asian population. As our study included a study population of both Asians and Caucasians, it indicates the generalizability of the study result. However, the study subjects were more from the Asian population. The lowest and highest average age among the cases was 36.3 and 78 years, respectively, and it was 35.5 and 77 years, respectively, among the controls in this study. Among cases, eight studies had male predominance, and only two studies had female predominance. In one of the studies, all the cases were of the male sex [13]. Five studies reported fibrinogen by taking cut-off value, while an equal number of studies had reported it separately for the case and control, respectively.

Methodological Quality of Study

The quality of these included studies was assessed by the Newcastle-Ottawa scale criteria (Table 3). Among these 10 studies, the highest score was 9 [13], and the lowest score was 7 [7,11,14,15] on the Newcastle-Ottawa scale. Out of these, seven studies were of good quality and three of fair quality as per AHRQ standards.

**Table 3:** Newcastle-Ottawa scale for judgment of the quality of individual study AHRQ std.: Agency for Health Care Research and Quality Standard. Source: References [7-16].

		Selection				Comparability		Exposure				AHRQ Std.
S. No.	Study	Adequate Case Definition	Selection of Representativeness of Case	Selection of Representativeness of Control	Definition of Controls	Study Controls for the Most Important Factor	Study Controls for Any Additional Factor	Ascertainment of Exposure (Max. of 2 Stars)	Same Method of Ascertainment for Case and Control	No Response Rate	Total	
1	Qizilbash et al., 1991 [7]	1	1	1		1	1	1	1		7	Good
2	Kristensen et al., 1998 [8]	1	1	1	1	1	1	1	1		8	Good
3	Bots et al., 2002 [9]	1	1	1	1	1	1	1	1		8	Good
4	Milionis et al., 2005 [10]	1	1	1	1	1	1	1	1		8	Good
5	Zhang et al., 2007 [11]	1	1		1	1	1	1	1		7	Fair
6	Jood et al., 2008 [12]	1	1	1	1	1	1	1	1		8	Good
7	Prugger et al., 2013 [13]	1	1	1	1	1	1	2	1		9	Good
8	Imran et al., 2015 [14]	1	1		1	1	1	1	1		7	Fair
9	Tao et al., 2020 [15]	1	1		1	1	1	1	1		7	Fair
10	Karim et al., 2020 [16]	1	1	1	1	1	1	1	1		8	Good

Diagnostic Accuracy

**Figure 2:**
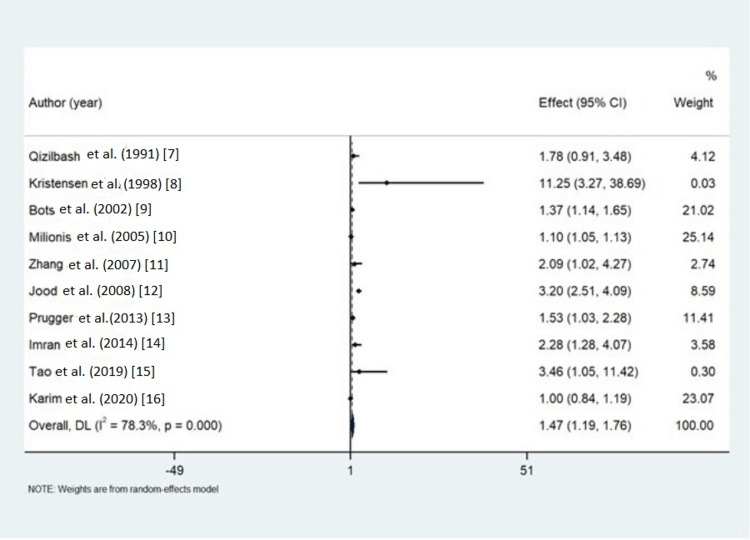
Forest plot with pooled odds ratio Source: References [7-16].

Outcomes of the studies were recorded in the form of pooled OR with upper and lower CIs as shown in the forest plot (Figure 2). The pooled OR was 1.47 with a CI of 1.19 to 1.76. The heterogeneity (I^2^) was 78.3% with a p-value of 0.00. As the heterogeneity was more than 50%, the random effect model was used.

Publication Bias

**Figure 3:**
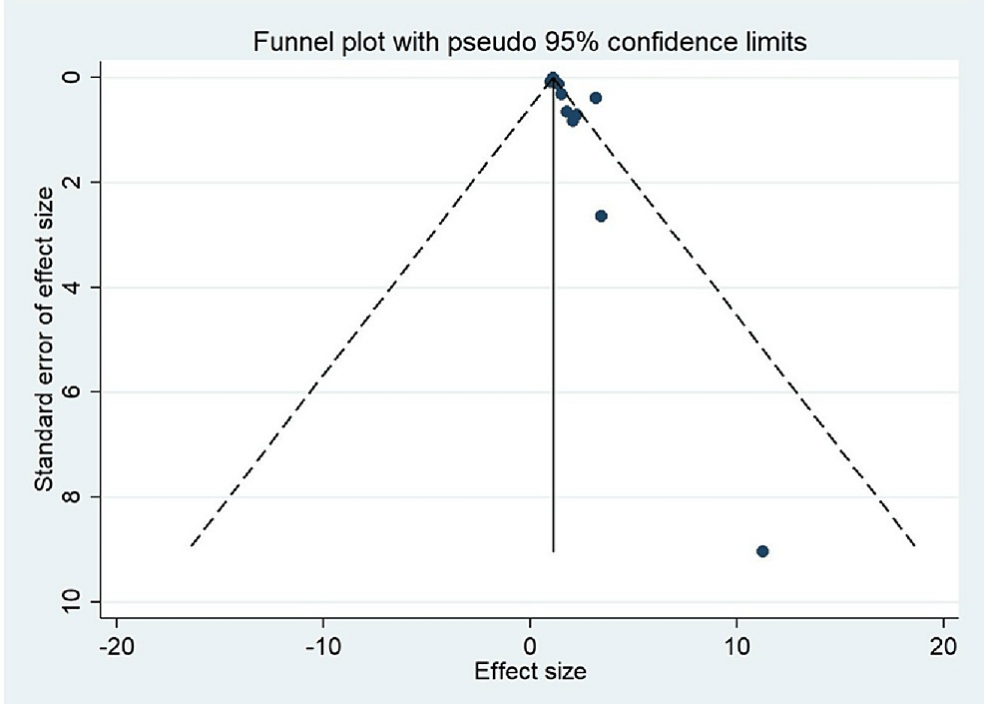
Funnel plot for assessing publication bias Image credits: Authors of this study.

 A funnel plot was drawn to search for publication bias (Figure 3), and it was found to be significant (p-value = 0.022).

Meta-regression Analysis

Important variables were also recorded in the studies, which can influence the study outcome. These variables were age, gender, diabetes, hypertension, smoking, alcohol, total cholesterol, serum triglyceride, fibrinogen cut-off level, sample size, ethnicity, BMI, HDL, and LDL (Table 2). Following meta-regression (bubble plot in Figures 4-17), none of these variables had an influence on the effect size as the individual p-value for these variables was >0.05 (Table 4).

**Figure 4:**
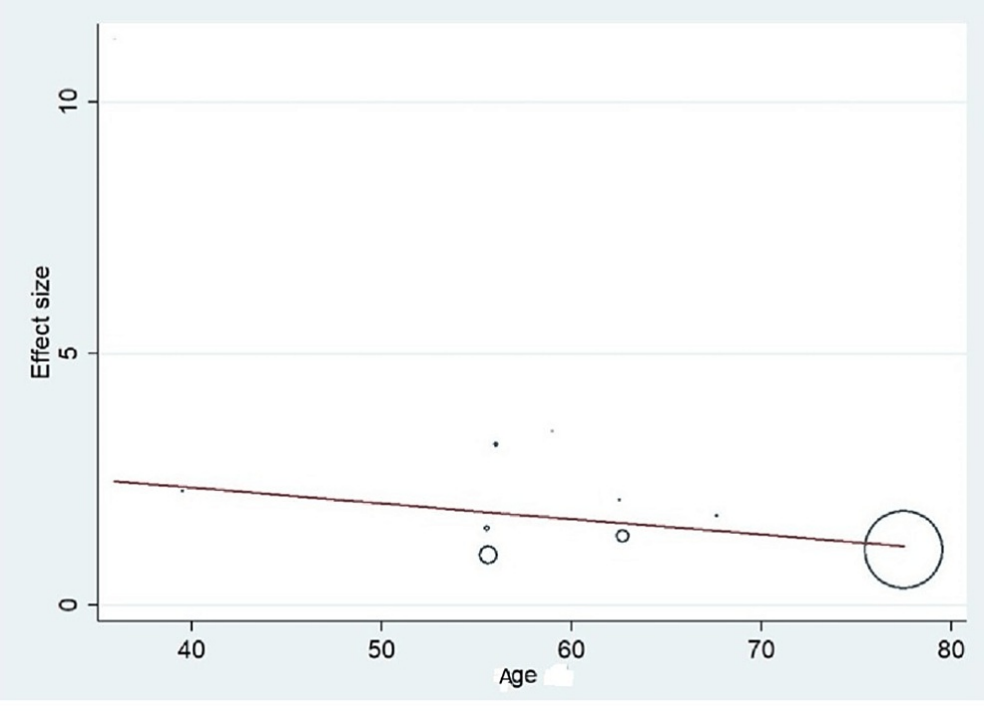
Bubble plot showing meta-regression analysis to determine the influence of age in the study population with the effect size This image is self-generated using the collected data from the individual studies using the command "metareg" in the software STATA version 13 to explore the source of heterogeneity due to variation in age.

**Figure 5:**
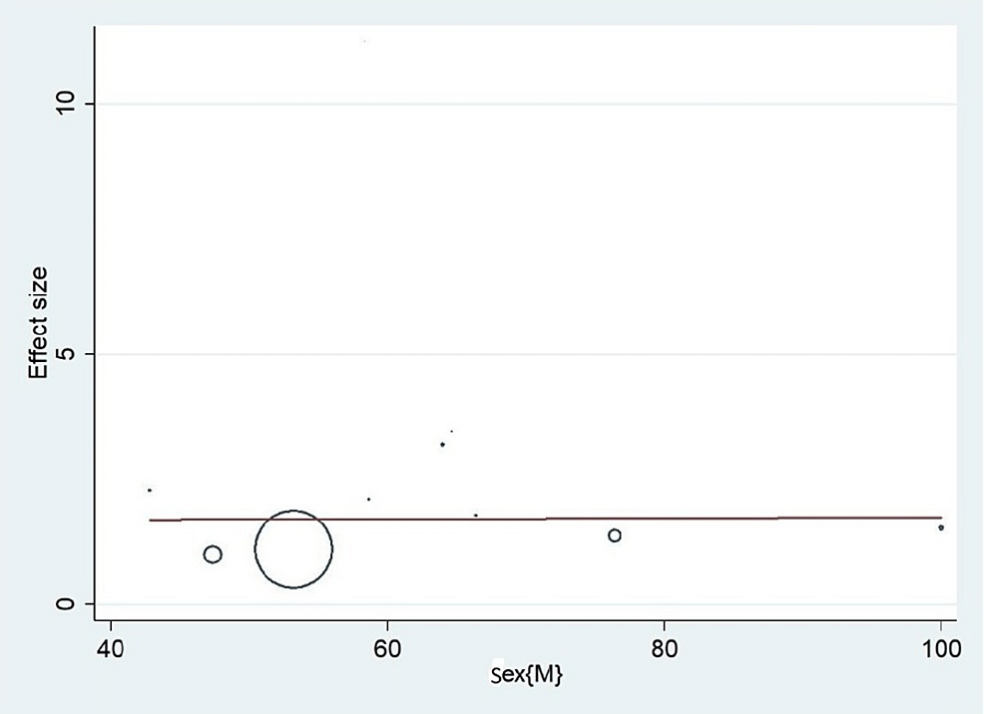
Bubble plot showing meta-regression analysis to determine the influence of gender in the study population with the effect size This image is self-generated using the collected data from the individual studies using the command "metareg" in the software STATA version 13 to explore the source of heterogeneity due to gender variability.

**Figure 6:**
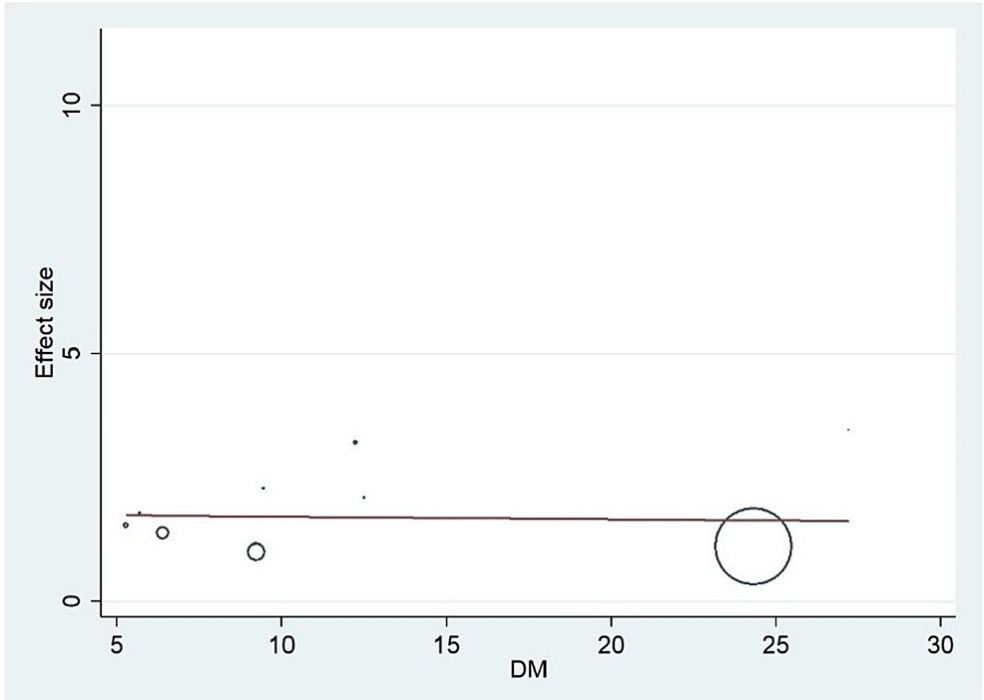
Bubble plot showing meta-regression analysis to determine the influence of diabetes in the study population with the effect size This image is self-generated using the collected data from the individual studies using the command "metareg" in the software STATA version 13 to explore the source of heterogeneity due to the presence of diabetes mellitus.

**Figure 7:**
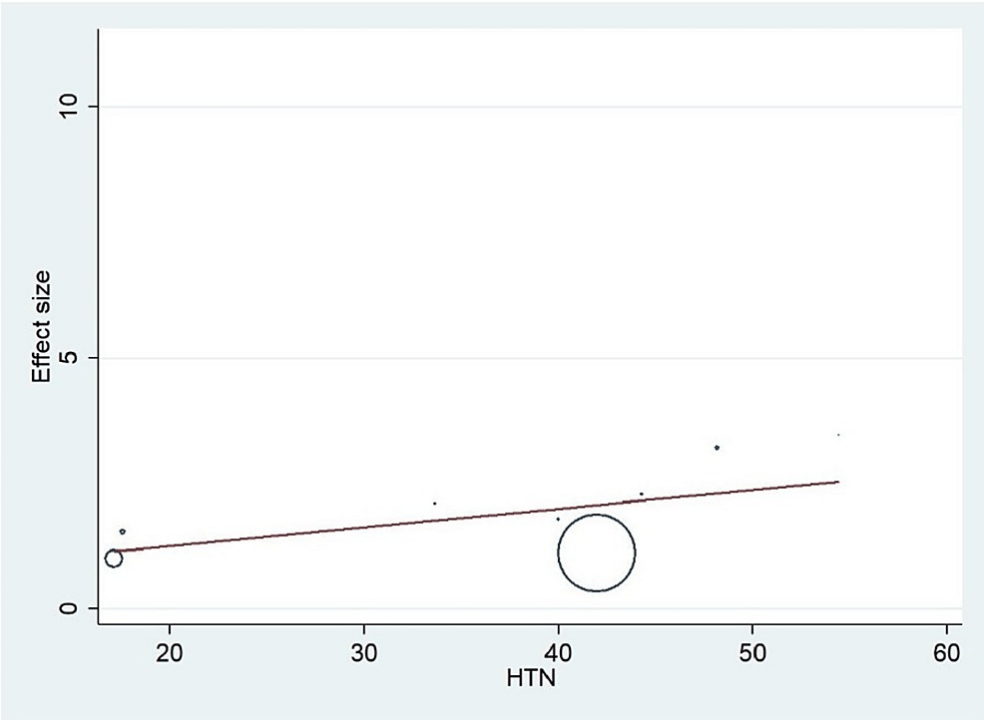
Bubble plot showing meta-regression analysis to determine the influence of hypertension (HTN) in the study population with the effect size This image is self-generated using the collected data from the individual studies using the command "metareg" in the software STATA version 13 to explore the source of heterogeneity due to the presence of hypertension.

**Figure 8:**
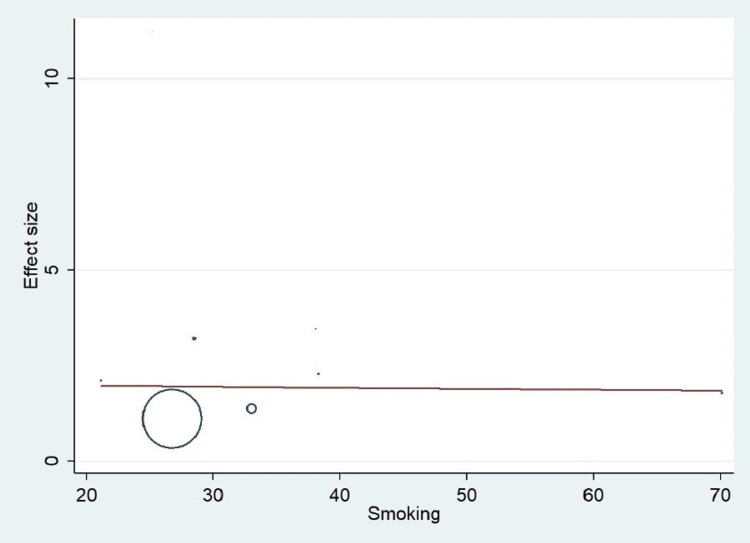
Bubble plot showing meta-regression analysis to determine the influence of smoking in the study population with the effect size This image is self-generated using the collected data from the individual studies using the command "metareg" in the software STATA version 13 to explore the source of heterogeneity due to the presence of smoking.

**Figure 9:**
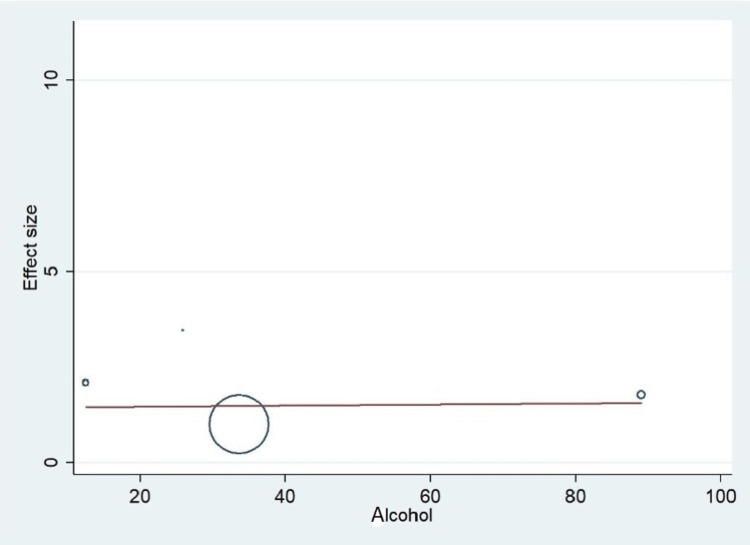
Bubble plot showing meta-regression analysis to determine the influence of alcohol in the study population with the effect size This image is self-generated using the collected data from the individual studies using the command "metareg" in the software STATA version 13 to explore the source of heterogeneity due to alcoholism.

**Figure 10:**
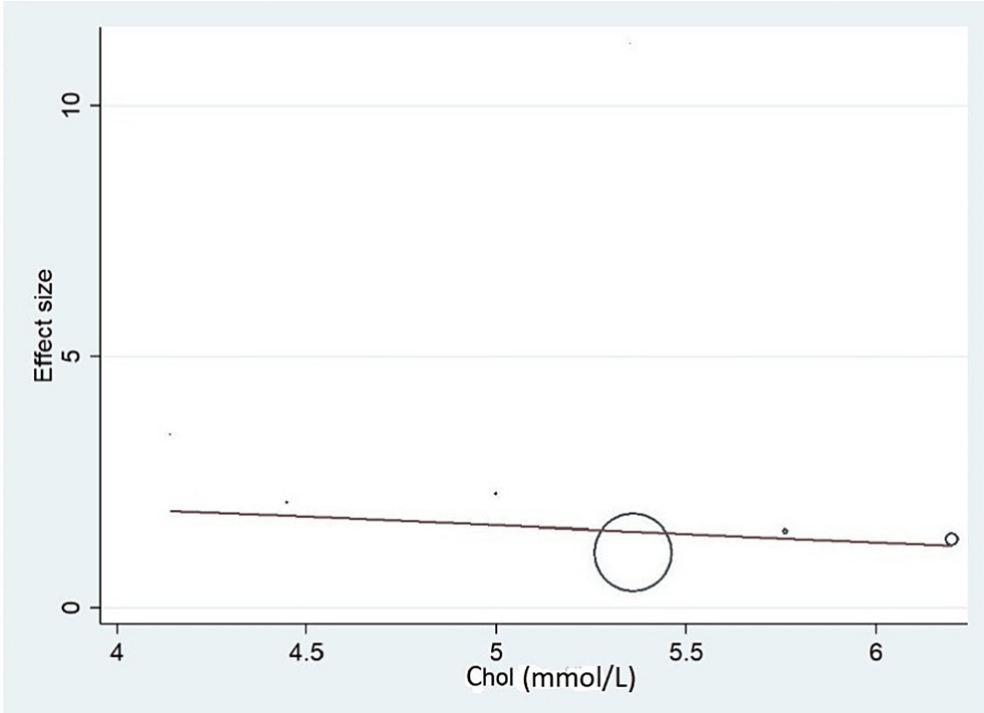
Bubble plot showing meta-regression analysis to determine the influence of total cholesterol in the study population with the effect size This image is self-generated using the collected data from the individual studies using the command "metareg" in the software STATA version 13 to explore the source of heterogeneity due to different levels of cholesterol.

**Figure 11:**
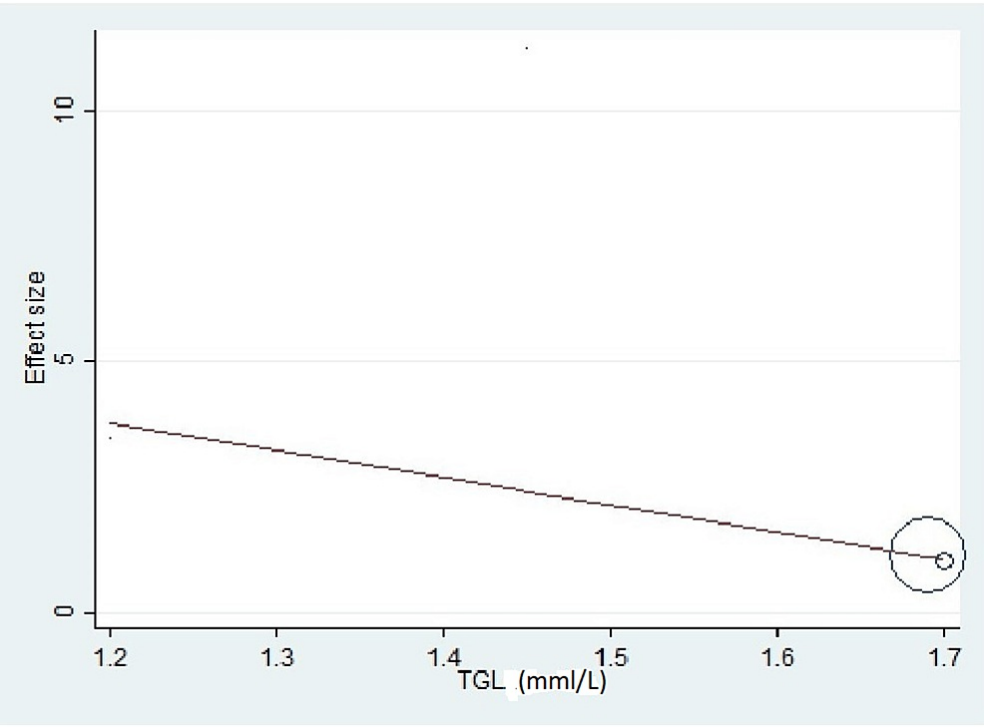
Bubble plot showing meta-regression analysis to determine the influence of triglyceride in the study population with the effect size This image is self-generated using the collected data from the individual studies using the command "metareg" in the software STATA version 13 to explore the source of heterogeneity due to different levels of triglyceride.

**Figure 12:**
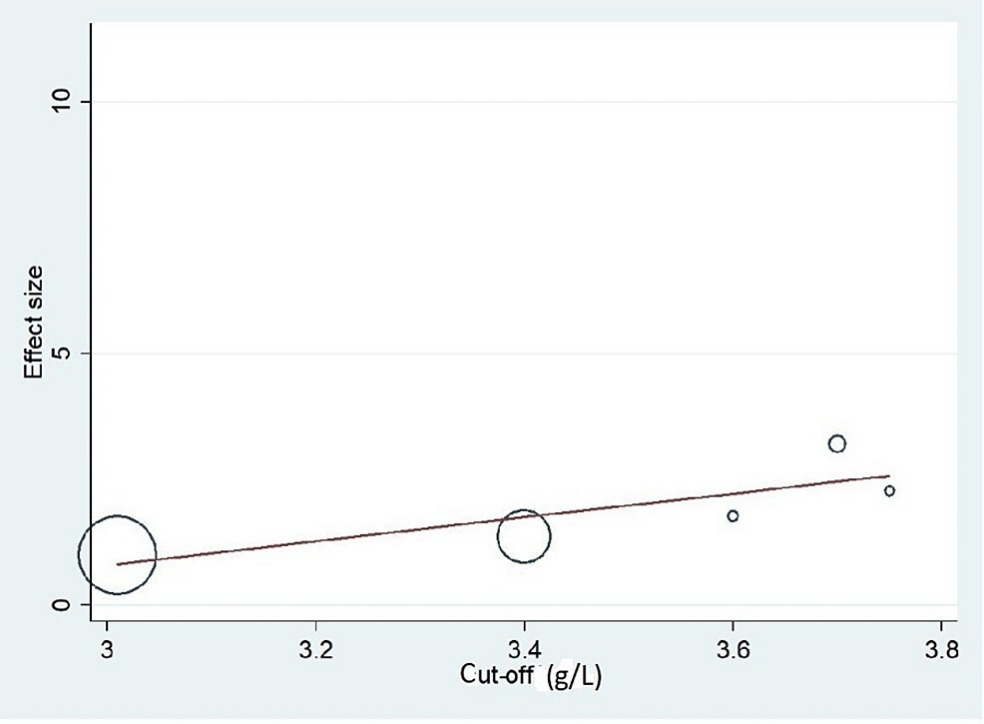
Bubble plot showing meta-regression analysis to determine the influence of cut-off (serum/plasma and fibrinogen level) in the study population with the effect size This image is self-generated using the collected data from the individual studies using the command "metareg" in the software STATA version 13 to explore the source of heterogeneity due to different cut-offs for fibrinogen.

**Figure 13:**
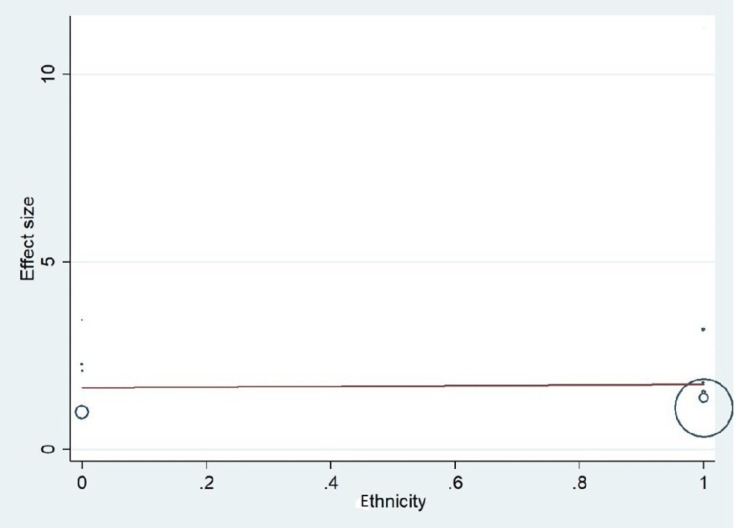
Bubble plot showing meta-regression analysis to determine the influence of ethnicity in the study population with the effect size This image is self-generated using the collected data from the individual studies using the command "metareg" in the software STATA version 13 to explore the source of heterogeneity due to different ethnicities.

**Figure 14:**
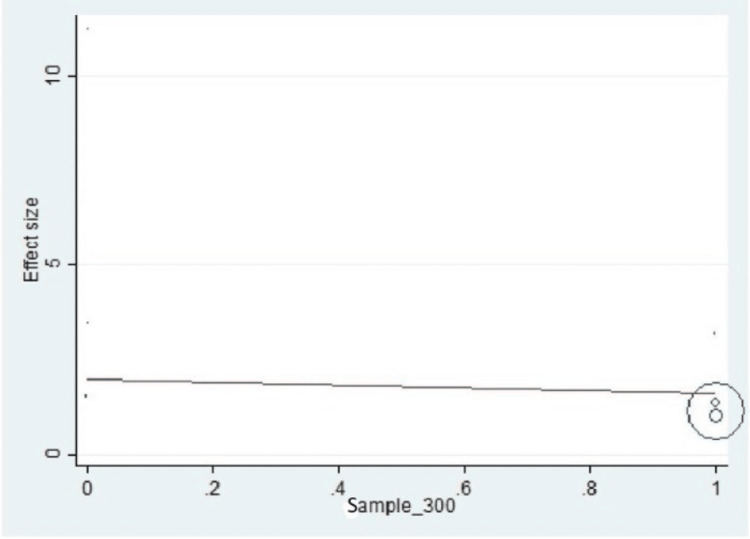
Bubble plot showing meta-regression analysis to determine the influence of sample size in the study population with the effect size This image is self-generated using the collected data from the individual studies using the command "metareg" in the software STATA version 13 to explore the source of heterogeneity due to different sample sizes.

**Figure 15:**
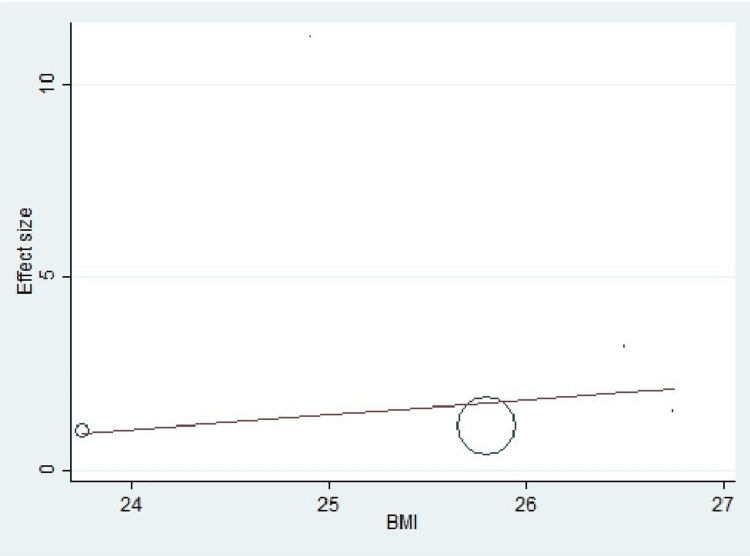
Bubble plot showing meta-regression analysis to determine the influence of body mass index (BMI) in the study population with the effect size This image is self-generated using the collected data from the individual studies using the command "metareg" in the software STATA version 13 to explore the source of heterogeneity due to different levels of BMI.

**Figure 16:**
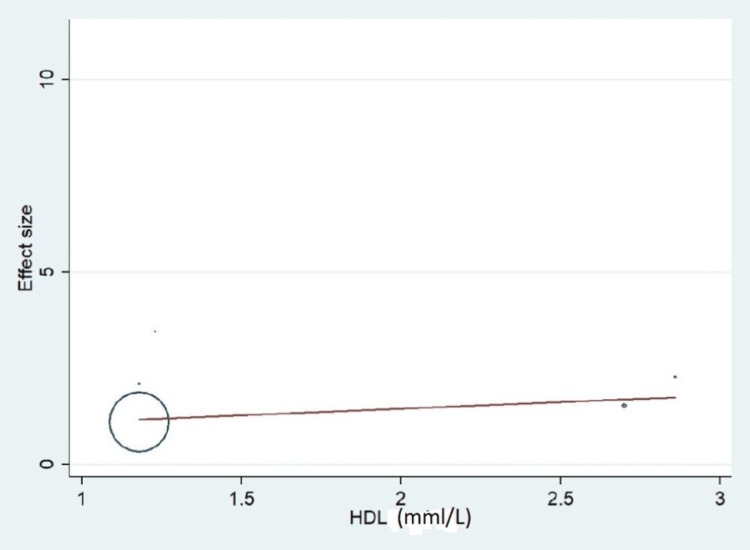
Bubble plot showing meta-regression analysis to determine the influence of high-density lipoprotein (HDL) in the study population with the effect size This image is self-generated using the collected data from the individual studies using the command "metareg" in the software STATA version 13 to explore the source of heterogeneity due to different levels of HDL.

**Figure 17:**
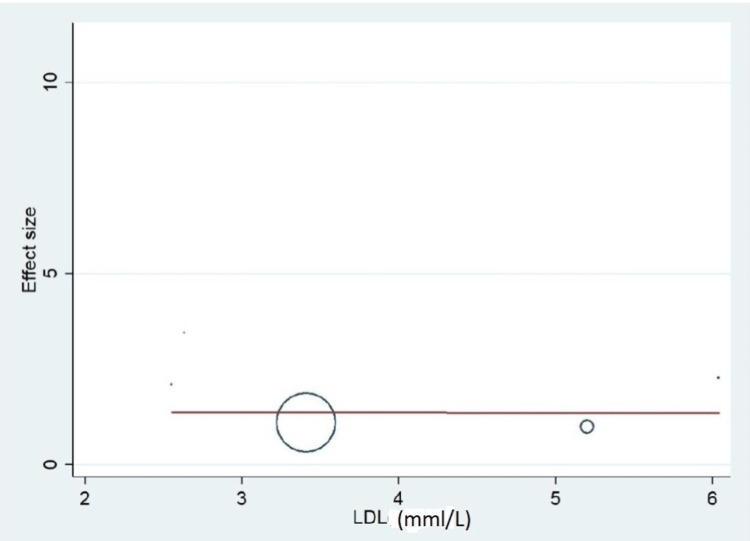
Bubble plot showing meta-regression analysis to determine the influence of low-density lipoprotein (LDL) in the study population with the effect size This image is self-generated using the collected data from the individual studies using the command "metareg" in the software STATA version 13 to explore the source of heterogeneity due to different levels of LDL.

**Table 4:** P-value after meta-regression for different variables BMI: Body mass index; HDL: High-density lipoprotein; LDL: Low-density lipoprotein.

S. No.	Variables	P-value
1	Age	0.288
2	Gender	0.968
3	Diabetes	0.906
4	Hypertension	0.165
5	Smoking	0.918
6	Alcohol	0.940
7	Total cholesterol	0.479
8	Triglyceride	0.068
9	Fibrinogen cut-off value	0.088
10	Ethnicity	0.88
11	Sample size	0.57
12	BMI	0.90
13	HDL	0.29
14	LDL	0.99

Discussion

There are many risk factors like diabetes, hypertension, smoking, and old age, which attribute to stroke. Fibrinogen is one such risk factor. A meta-analysis was done on fibrinogen as a risk factor for myocardial infarction in the cardiovascular system. In this meta-analysis, the OR found was 2.3 (CI: 1.9 to 2.8); this indicates that serum fibrinogen level is associated with an increased risk of cardiovascular events [4]. The fibrinogen collaboration studies found that there is an approximate double risk of major cardiovascular events when there is a long-term elevation of serum fibrinogen level of 1 gm/liter [17].

As fibrinogen plays a principal role in cardiovascular events, it must have a role in ischemic stroke as the pathophysiology of myocardial infarction and ischemic stroke is almost the same. Till now, no meta-analysis has been done to look for the association of fibrinogen as a risk factor for ischemic stroke. Our meta-analysis having 10 studies used vigorous statistical procedure and methodological quality to obtain inferences about fibrinogen as a risk factor for ischemic stroke. Our meta-analysis having pooled OR of 1.47 (CI: 1.19-1.76) gives an inference that patients with raised fibrinogen levels are 1.47 times more likely to have an increased risk of ischemic stroke. The heterogeneity (I^2^) in our study is 78.3% (p = 0.00), which is significant and may be due to the publication bias as shown by the funnel plot (Figure 3) with a p-value of 0.022. Another cause of heterogeneity may be due to a lack of blinding to case/control status in the structured interview for ascertainment of exposure as per our quality of study assessment by the Newcastle-Ottawa scale (Table 3). This high heterogeneity may be because all studies included in our meta-analysis are of case-control design. The research population included is of both Asians and Caucasians, which represents the generalizability of the study.

Following meta-regression for different variables (age, gender, diabetes mellitus, hypertension, smoking, alcohol, total cholesterol, serum triglyceride, fibrinogen cut-off level, ethnicity, sample size, BMI, LDL, and HDL) in our meta-analysis, none of these variables had influenced the effect size. Hence, fibrinogen may be a predictor for an increased risk of ischemic stroke.

The strength of our study is that it is supposed to be the first meta-analysis that has individually looked for the association of fibrinogen as a risk factor for ischemic stroke. We have reported our meta-analysis as per the latest PRISMA protocol 2020. This systematic review and meta-analysis are very much in line with the contemporaneous literature of interest.

However, there are certain limitations to our study. Only one of our studies had more than 10,000 study participants, and the remaining nine studies had study participants ranging from 100 to 1200. All of these studies were case-control studies, so in the future, we will need cohort studies with larger sample sizes and a greater number of studies to validate the association of fibrinogen as a risk factor for ischemic stroke. Different studies included in our meta-analysis have reported serum/plasma fibrinogen levels in different types of ischemic stroke ranging from minor to major ischemic stroke, so it is difficult to say whether the increased level of fibrinogen is associated more with minor or major ischemic stroke. As we have included different studies published in the English language only, there can be language bias. Another limitation is that we have not seen the correlation between fibrinogen level and the incidence of ischemic stroke.

## Conclusions

Our meta-analysis concluded that increased fibrinogen levels are associated with an increased risk of ischemic stroke. However, the role of fibrinogen as a risk factor for ischemic stroke is ambiguous due to the presence of significant heterogeneity and publication bias. But, it may be used to identify those at serious risk for ischemic stroke and then to initiate ischemic stroke intervention.

So, we conclude this discussion with the hope that, in the future, a greater number of high-quality studies, including individual participant meta-analyses with larger sample sizes, will be conducted in order to accept increased fibrinogen levels as a risk factor for ischemic stroke.
